# Supplementary material for: Intelligent Coatings with Controlled Wettability for Oil–Water Separation
Source: Nanomaterials (Basel). 2022 Sep 8;12(18):3120. doi: 10.3390/nano12183120 (PMC9500904; doi:10.3390/nano12183120)
Supplement: Supplementary file 1 [file nanomaterials-12-03120-s001.zip › nanomaterials-1881471-supplementary.pdf]

soybean oil and water.mp4

Video S1. The separation process for soybean oil/water mixture

tetrachloromethane and water.mp4

Video S2. The separation process for tetrachloromethane/water mixture
